# Effects of soil amendment with PCB-contaminated sediment on the growth of two cucurbit species

**DOI:** 10.1007/s11356-019-06509-9

**Published:** 2019-11-04

**Authors:** Magdalena Urbaniak, Sunmi Lee, Mari Takazawa, Elżbieta Mierzejewska, Agnieszka Baran, Kurunthachalam Kannan

**Affiliations:** 1 Wadsworth Center, New York State Department of Health, Empire State Plaza, P.O. Box 509, Albany, NY 12201-0509 USA; 2 Department of Applied Ecology, Faculty of Biology and Environmental Protection, University of Lodz, Banacha 12/16, 90-237 Lodz, Poland; 3 Faculty of Agriculture and Economics, Department of Agricultural and Environmental Chemistry, University of Agriculture in Krakow, Al. Mickiewicza 21, 31-120 Krakow, Poland; 4 Department of Environmental Health Sciences, School of Public Health, State University of New York at Albany, New York, NY USA

**Keywords:** PCBs, Hudson River, Sediment, Cucurbits, Phytoremediation, Rhizoremediation, Plant condition

## Abstract

The aim of the study was to evaluate the influence of the application of increasing proportions (0%, 10%, 25%, 50%, 75%, and 100%) of an admixture of PCB-contaminated Hudson River sediment collected from the Upper Hudson River, near Waterford, Saratoga county (New York, USA) on soil properties, phytotoxicity, and biometric and physiological responses of cucumber (*Cucumis sativus* L. cv ‘Wisconsin SMR 58’) and zucchini (*Cucurbita pepo* L. cv ‘Black Beauty’) grown as potential phyto- and rhizoremediators. The experiment was performed for 4 weeks in a growth chamber under controlled conditions. Amendment of Hudson River sediment to soil led to a gradual increase in PCB content of the substratum from 13.7 μg/kg (with 10% sediment) to 255 μg/kg (with 100% sediment). Sediment amendment showed no phytotoxic effects during the initial stages, even *Lepidium sativum* root growth was stimulated; however, this positive response diminished following a 4-week growth period, with the greatest inhibition observed in unplanted soil and zucchini-planted soil. The stimulatory effect remained high for cucumber treatments. The sediment admixture also increased cucurbit fresh biomass as compared to control samples, especially at lower doses of sediment admixture, even though PCB content of the soil amended with sediment increased. Cucurbits’ leaf surface area, in turn, demonstrated an increase for zucchini, however only for 50% and 75% sediment admixture, while cucumber showed no changes when lower doses were applied and decrease for 75% and 100% sediment admixture. Chlorophyll *a* + *b* decreased significantly in sediment-amended soils, with greater inhibition observed for cucumber than zucchini. Our results suggest that admixture of riverine sediment from relatively less-contaminated locations may be used as soil amendments under controlled conditions; however, further detailed investigation on the fate of pollutants is required, especially in terms of the bioaccumulation and biomagnification properties of PCBs, before contaminated sediment can be applied in an open environment.

## Introduction

The Hudson River flows for 507 km from its source in the Adirondack Mountains to Southern Manhattan, New York (NY), USA. The river is a cultural, social, commercial, recreational, and ecological resource for millions of people inhabiting the watershed (Pinkney et al. 2017). Between 1947 and 1977, large quantities of polychlorinated biphenyls (PCBs) were released into the upper section of the river from the capacitor-manufacturing operations at the General Electric (GE) plants at Hudson Falls and Fort Edward, NY. It is estimated that during that time, at least 590,000 kg of PCBs was released into the river (www.epa.gov/hudson/). PCBs bound to sediment particles pose a serious threat to this riverine ecosystem and human health. The removal of Fort Edward Dam in 1973 allowed for even greater discharge of PCB-contaminated sediments downstream, creating additional risk for biota and local communities. This resulted in a reduction in ecological and recreational value of the Hudson River, and prohibition of various activities, including recreational fishing (Hudson River Natural Resource Trustees 2015).

In 1984, the U.S. Environmental Protection Agency (US EPA) designated the nearly 322-km section of the Hudson River from Hudson Falls to the southern tip of Manhattan as a Superfund Site (US EPA 2002). The site is divided into two parts: the Upper Hudson River, which covers the area from Hudson Falls to the Federal Dam at Troy (approximately 64 km); and the tidal Lower Hudson River, covering the river section from the Federal Dam at Troy to the southern tip of Manhattan. In February 2002, the EPA finalized a Record of Decision for the Hudson River PCB Superfund Site that called for targeted environmental dredging of approximately 2.10 million m^3^ of PCB-contaminated sediment from a 64-km section of the Upper Hudson River (www.epa.gov/hudson/). Dredging started in 2009 and was finalized in 2015, resulting in the removal of estimated 141,000 kg of PCBs (US EPA 2015). Un-dredged PCB residues, however, still constitute a threat for the river itself and the surrounding ecosystems, including the floodplain soil and groundwater, as well as the organisms that inhabit the ecosystem.

Remediation of the remaining PCB-contaminated sediments in situ poses many technological and logistical problems (Cho et al. 2001). The dredged sediment can be disposed in remote sites; however, PCB concentrations can be further reduced during or before disposal, with the use of plants (rhizo- and phytoremediation). Due to their hydrophobicity, and hence their strong adsorption to sediment and soil particles, PCBs only sparingly transfer to solution, and as a consequence, most plants only have limited potential to take up PCBs from sediment or soil (Briggs et al. 1982; Terzaghi et al. 2018b). One notable exception is the *Cucurbitaceae* family, including zucchini and cucumber, which readily takes up hydrophobic compounds, such as PCBs, from soil and translocates them to leaves and fruits (Greenwood et al. 2011; Matsuo et al. 2011; Low et al. 2011). It has been demonstrated that the members of *Cucurbitaceae* can phytoextract other organic compounds, including PCDDs/PCDFs (Engwall and Hjelm 2000; Hülster et al. 1994; Urbaniak et al. 2016), *p*,*p*′-DDE (White 2002), DDT (Aslund et al. 2010), PAHs (Parrish et al. 2006), and chlordane (Mattina et al. 2000). Plants, including cucurbits, also play a key role in soil ecosystems by stabilizing soil structure, and stimulating soil microbial activity by serving as primary sources of organic matter and energy (Machado Vezzani et al. 2018). Moreover, due to existing interactions between plant roots, root exudates, soil, and microorganisms, microbe-assisted phytoremediation (rhizoremediation) is thought to be the most effective method for the remediation of contaminated soil. Therefore, the use of cucurbits and associated rhizosphere bacteria appears to be a promising approach for the remediation of riverine sediments polluted with PCB mixtures and most likely other organic pollutants.

The overall aim of this study was to evaluate the suitability of sediment substrate amended to soils for the growth of common cucurbits, cucumber (*Cucumis sativus* L. cv ‘Wisconsin SMR 58’) and zucchini (*Cucurbita pepo* L. cv ‘Black Beauty’). The influence of the river sediment on PCB content and phytotoxicity of soil–sediment mixtures were measured using Phytotoxkit and by growing cucumber and zucchini for 4 weeks at various doses of sediments amended to soil. In addition, to assess the effect of PCB-contaminated river sediments on biometric and physiological parameters of cucumber and zucchini, total aboveground biomass, leaf surface area, and chlorophyll *a* + *b* content were determined.

## Materials and methods

### Materials

#### Soil

The vegetable potting soil was obtained from FoxFarm Soil & Fertilizer Company (Arcata, California, USA). The soil composition was described as follows: aged forest products, sphagnum peat moss, Pacific Northwest Sea-going fish emulsion, crab meal, earthworm castings, sandy loam, perlite, fossilized bat guano, granite dust, Norwegian kelp meal, and oyster shell. The physico-chemical properties and PCB content of soil used in the experiment were determined (see section on soil and sediment physical and chemical properties), and the results are presented in Table **1**.

**Table 1:** Physical and chemical properties of potting soil and Hudson River sediment collected above Waterford and used in the experiment

Properties	Vegetable potting soil	Hudson River sediments	TEC (threshold effect concentration)	PEC (probable effect concentration)
pH in KCl	5.26	7.38	–	–
pH in H_2_O	5.60	6.88	–	–
Salinity (mS)	0.88	3.21	–	–
TDS (g/L)	0.45	1.61	–	–
Macroelements				
TOC (%)	36.4	1.69	–	–
N (%)	0.84	0.14	–	–
S (%)	0.27	0.06	–	–
Ca (g/kg)	11.0	2.24	–	–
K (g/kg)	1.28	1.14	–	–
Mg (g/kg)	2.51	1.78	–	–
P (g/kg)	0.87	**4.01**	–	–
Na (mg/kg)	351	140	–	–
Fe (g/kg)	–	**3.06**	–	–
Heavy metals				
Cd (mg/kg)	0.66	0.28	0.99	4.98
Cr (mg/kg)	7.67	4.13	43.3	111
Cu (mg/kg)	9.86	6.48	31.6	149
Mn (mg/kg)	130	89.5	–	–
Ni (mg/kg)	3.44	2.88	22.7	48.9
Pb (mg/kg)	4.25	1.95	35.8	128
Zn (mg/kg)	53.7	15.4	123	459
Organic compounds				
Total PCBs (μg/kg)	0.41	255	59.8	676

#### Riverine sediments

Riverine sediments were collected at a location upstream of Waterford village, at the Upper Hudson River Near Waterford (WTFN6) gauge station (Saratoga county, NY, USA) located above the Mohawk River tributary and above Champlain Canal Lock 1 (Lock One Rd., Waterford, NY). The sampling site (coordinates 42°49′36.9″N 73°39′55.2″W) is located in the area of the Upper Hudson River PCB Superfund Site (www.epa.gov/hudson/). The hydrological parameters of the Hudson River at WTFN6 during the sampling period were as follows: flow 493 m^3^/s, water stage 7 m. Based on information from the National Weather Service—Advanced Hydrologic Prediction Service (www.water.weather.gov), the flood stage at the WTFN6 gauge station was 10.4 m. Sediment samples were collected during stable hydrologic conditions. Sediment samples were collected using a grab sampler and transported to the laboratory where they were used as a vegetable potting soil additive/amendment. The physico-chemical properties of the sediment were determined (see section on soil and sediment physical and chemical properties**)** and the results depicted in Table 1.

#### Plants

Seeds of cucumber (*Cucumis sativus* L. cv ‘Wisconsin SMR 58’; purchased from Seeds’nSuch, Graniteville, South Carolina, USA) and zucchini (*Cucurbita pepo* L. cv ‘Black Beauty’; purchased from Botanical Interests, Inc., Broomfield, Colorado, USA) were germinated in Petri dishes for 4 days to obtain seedlings of the same growth stage. Selected seedlings of the same size and at the same growth phase were planted in soil–sediment mixtures as described below.

### Experimental setup

The vegetable potting soil was mixed with fresh Hudson River sediment, in the following proportions: 0%, 10%, 25%, 50%, 75%, and 100% river sediment. The proportion of soil and sediment was calculated based on their dry weight. To obtain homogenized samples, the sediment was mixed in a glass dish for 1 h using a stainless steel spatula; a similar procedure was adopted for vegetable potting soil. In order to prepare the soil–sediment mixtures, river sediment and vegetable potting soil were thoroughly mixed for approximately 1 h using a stainless steel spatula. Each experiment was prepared in nine replicates in a polypropylene pot (capacity 400 cm^3^). Three replicates were incubated without plants; three were planted with cucumber and three with zucchini seedlings, with three seedlings per replicate (pot): nine seedlings in total per single treatment. The unplanted and planted (soil/sediment) samples were incubated for 4 weeks (28 days) in a growth chamber (Thermo Scientific Plant Growth Chamber 3768, Marietta, Ohio, USA) at 25 ± 0.5 °C with a 16-h light/8-h dark cycle, and 150± 5 μmol m^−2^ s^−1^ photon flux density during the light period. All variants (planted and unplanted) were watered daily: the planted variants with 15–25 mL of water (depending on the plant’s growth stage) and the unplanted variants with 15 mL.

### Physical and chemical analysis of soil and sediment

Samples of the soil and sediment mixture were air dried, and a Vario Max Cube analyzer was used to measure organic carbon, nitrogen, and sulfur content. In addition, trace elements were determined using an Optima 7300 DV inductively coupled plasma optical emission spectrophotometer (ICP-OES). The total concentrations of macroelements Na, Mg, Ca, K, P, and N and heavy metals Fe, Mn, Ni, Cr, Zn, Pb, and Cd were determined after digestion in a mixture of HNO_3_ and HClO_3_ (3:2 *v*/*v*).

### Soil–sediment mixture analysis

#### PCB analysis

Soil–sediment mixture from each variant (in three replicates, each 400 cm^3^) was thoroughly mixed and homogenized using a stainless steel spatula in order to obtain one representative sample. A 100-g subsample was collected and freeze dried. After this step, the dried sample was again homogenized in a ceramic mortar, and subsample of 2 g was collected and subjected for analysis of PCB congeners (di-CBs—PCB-10/4, 6, 5/8, 15; tri-CBs—PCB-19, 17/18, 26, 31/28, 33/20, 22, 37; tetra-CBs—PCB-53, 51, 45, 52/73, 49/43, 44, 41, 64, 63, 74/61, 70/76, 60/56, 77; penta-CBs—PCB-93/95, 92/84, 90/101/89, 99, 119, 9/113, 110, 82, 123, 11/106, 114/122, 105/127; hexa-CBs—PCB-136, 151, 135/144, 149/139, 134, 146, 161, 153, 141, 137, 130, 138/164/163, 156, 157, 169; hepta-CBs—PCB-187/182, 183, 174, 177, 171, 180, 193, 190; octa-CBs—PCB-202, 201, 196/203, 95, 194, 205; nona-CBs—PCB-208 and 206; and deca-CB—PCB-209).

The sample was spiked with ^13^C-labeled internal standard (IS) mixture (PCB-LCS-H, 20 ng each; Wellington Laboratories Inc., Guelph, Ontario, Canada) and extracted with hexane by an Accelerated Solvent Extractor (ASE 200; Dionex, Sunnyvale, California, USA) at 1500 psi, 100 °C. The samples were purified by passage through a multilayer silica column packed with neutral and acidic silica gel and elution with hexane. The extracts were further concentrated to 500 μL under a gentle stream of nitrogen. The identification and quantification of PCB congeners were performed by Agilent Technologies 7890B GC coupled with 5977B MSD connected to a Zebron 5MS (15 m, 0.25 mm i.d., 0.10 μm film thickness; Phenomenex, Torrance, California, USA) capillary column. The GC was operated in the split-less injection selected ion monitoring (SIM) mode. A 12-point calibration curve with concentrations that ranged from 0.05 to 200 ng/mL was used to determine concentrations.

QA/QC protocols include IS method of quantification using certified calibration standards and labeled IS. Each analytical batch contained two procedural blanks, two matrix spikes (native standards spiked at 20 ng), and replicate analysis of samples. Recoveries ranged from 88.9% to 101% for di-CBs, from 78.6% to 119% for tri-CBs, from 92.4% to 118% for tetra-CBs, from 83.3% to 92.1% for penta-CBs, from 85.7% to 102% for hexa-CBs, from 79.2% to 96.8% for hepta-CBs, from 82.6% to 97.7% for octa-CBs, from 67.3% to 88.9% for nona-CBs, and 88.3% for deca-CB. Limit of quantitation was 0.05 μg/kg.

#### Phytotoxicity bioassay

The phytotoxicity of soil and soil–sediment mixture was assessed using a Phytotoxkit, a commercial toxicity bioassay kit (Environmental Bio-Detection Product Inc., Mississauga, Ontario, Canada) (Phytotoxkit 2004). The test measured inhibition of root length of the test species, *Lepidium sativum* (L.), after 72 h of exposure to soil–sediment mixtures, in relation to root length of *L. sativum* exposed to uncontaminated, reference OECD soil delivered along with the test kit. The response of the test species was classified as toxic when the root length inhibition was ≥ 20% (Persoone et al. 2003).

### Plant analysis

#### Fresh biomass measurement

The fresh biomass of cucumber and zucchini plants was measured after a 4-week (28 days) cultivation period. The plants were cut 1 cm above the soil surface and the fresh biomass of the above-ground parts was weighed.

#### Leaf surface area measurement

The leaves from each plant were collected after a 4-week (28 days) cultivation period and immediately scanned (within maximum 15 min after collection of each leaf to avoid losing of leaf turgor and shape that may influence on the measurement appropriateness) using a standardized template. The surface area of each leaf was measured using the ImageJ software package.

#### Determination of chlorophyll content

Chlorophyll content was measured in 4-week-old plants using the acetone method (Porra et al. 1989). Fresh leaves (0.2 g) of cucumber and zucchini were cut and homogenized in an ice-cold mortar. The crude homogenate obtained after filtration was assayed for chlorophyll content. The absorbance of a clear supernatant extract was measured at 663 and 645 nm using a Beckman DU 640 UV–Vis spectrophotometer.

### Statistical analysis

Statistica 12.0 software and Microsoft Excel were used for all statistical analyses. The differences between the means were analyzed by two-way ANOVA and Tukey’s test. The possible relationships between PCB concentration and physiological parameters of plants were evaluated using Pearson’s correlation. Significance was determined based on a probability score of *p* < 0.05.

## Results and discussion

### Physico-chemical properties of soil and sediment medium

Analyses of sediment properties are important for determining their potential as a soil amendment (Urbaniak et al. 2016; Tarnawski et al. 2017). For economic and ecological reasons, uncontaminated or less-contaminated dredged sediments are recommended for use in agriculture, especially for the cultivation of non-edible crops (Macía et al. 2014). Such agricultural utilization takes advantage of the beneficial properties of sediments, which are rich in clay, silt, organic matter, nutrients, and microbial activity (Karanam et al. 2008; Baran et al. 2010, 2012; Mattei et al. 2017). The Hudson River sediment collected at Waterford was neutral in pH (pH_KCl_ 6.88), and rich in minerals, as measured by electrical conductivity (3.21 mS/cm) (Table 1). A neutral or alkaline sediment is a suitable amendment for acidic soils. Moreover, higher pH is associated with lower mobility of toxic heavy metals (Baran et al. 2012). Our sediments were relatively low in total organic carbon (TOC), nitrogen (N), and sulfur (S) (Table 1).

The ratio of C/N has a direct influence on residue decomposition and nitrogen cycling, with greater decomposition occurring at lower ratios. In most soils, C/N ratio lies in the range of 8:1 to 10:1, whereas a ratio > 30 can result in N deficiencies. In our sediments, the average ratio of C/N was 12, which suggested that N is available for microbiological decomposition of organic matter. The sediments were rich in phosphorus (P) and calcium (Ca) but deficient in magnesium (Mg), potassium (K), and sodium (Na) (P > Ca > Mg > K > Na) (Table 1).

The concentrations of heavy metals in sediment were assessed using sediment quality guideline values referred as threshold effect concentration (TEC) and probable effect concentration (PEC) (MacDonald et al. 2000). Comparison of soil–sediment data with TEC indicated that collected sediments were non-toxic, as concentrations of metals were below the following thresholds: 123 mg/kg for Zn, 31.6 mg/kg for Cu, 35.8 mg/kg for Pb, 0.99 mg/kg for Cd, 22.7 mg/kg for Ni, and 43.3 mg/kg for Cr. Similarly, sediment is predicted to be toxic when exceeding the following PEC values: 459 mg/kg for Zn, 149 mg/kg for Cu, 128 mg/kg for Pb, 4.98 mg/kg for Cd, 48.6 mg/kg for Ni, and 111 mg/kg for Cr. None of the metals analyzed in sediments exceeded the TEC or PEC values.

PCB content was 0.410 μg/kg in vegetable potting soil and 255 μg/kg in sediment. The sediment PCBs concentration can be regarded as “low to moderate contamination” as it exceeded the TEC (3.8-fold) but it was below the PEC (Table 1). The same is true when comparing values obtained with the newest studies from the Hudson River, e.g., Xu et al. (2019) report a PCB concentration of 1590 μg/kg in analyzed sediment, this being more than 6-fold higher than in our study, whereas Rodenburg and Ralston (2017) showed total PCB concentrations (sum of 132 PCB) ranging from 2.3 ng/g to 361 μg/g d.w. Our PCB content of 255 μg/kg is, however, much lower than historical data: Huan Feng et al. (1998) report PCB concentrations between 80 and 1290 μg/kg, Achman et al. (1996) between 300 and 10 mg/kg, Carchich and Tofflemiere (1982) as high as 50 mg/kg, and Nadeau and Davis (1976) note concentrations as high as 6700 mg/kg.

Relatively high P and Fe content and low toxic metal content (below TEC level) suggested that the sediment may be used as a soil amendment. However, the sediment was poor in TOC, N, and S, which requires amendments of macroelements for growing plants. Nonetheless, our results do not reflect the quality and agricultural value of the sediments, as dispersal of sediments on a vast land can lead to dissipation of PCBs and other contaminants into the environment. Our goal was to test to the phytotoxicity and suitability of sediment as an amendment for use in a confined facility. The open use of contaminated sediment may lead to the dispersal of PCBs in the environment, even if the PCB content was below the PEC. Consequently, further detailed study on the fate of PCBs after application to the environment is required. It is also important to note that sediments used for this experiment do not reflect the pollution status of the entire Hudson River. Collection of sediments from different sections of the river would provide more comprehensive information on the suitability of sediments as soil admixture.

### The effect of sediment and cucurbit growth on soil PCB content

PCBs have low solubility in water and a strong tendency to adsorb on organic carbon; therefore, the most contaminated environmental matrices are soil and sediments. It is estimated that approximately 25% of the 1338 Superfund Sites in the USA (https://www.epa.gov/superfund/superfund-national-priorities-list-npl) are contaminated with PCBs. One of the most contaminated sites is the Hudson River Superfund Site (US EPA 2002), which is polluted with PCBs at concentrations ranging from nanograms per kilogram to hundreds of milligrams per kilogram in a number of locations (Xu et al. 2019; Rodenburg and Ralston 2017; Huan Feng et al. 1998; Carchich and Tofflemiere 1982; Achman et al. 1996; Nadeau and Davis 1976).

Several methods of PCB removal from sediments have been proposed. The most frequently used remediation technologies include dredging, dewatering, physical and chemical treatment (such as incineration), and landfilling. Since such approaches entail extensive costs, research is currently focused on the implementation of other more cost-effective and environmentally friendly remediation methods. One such method is phytoremediation, a method of pollutant removal based on the use of plants, especially those belonging to the cucurbit family, which are capable of taking up organic compounds from soil (Greenwood et al. 2011; Matsuo et al. 2011; Low et al. 2011; Engwall and Hjelm 2000; Hülster et al. 1994; White 2002; Aslund et al. 2010; Parrish et al. 2006; Mattina et al. 2000). Although certain plant species display good phytoextraction potential, the most effective method for the remediation of contaminated soil is thought to be rhizoremediation, a naturally occurring process within the plant root zone (rhizosphere), where the growth of microorganisms and their degradative activity are stimulated by root exudates (Mackova et al. 2006).

**Fig. 1:**
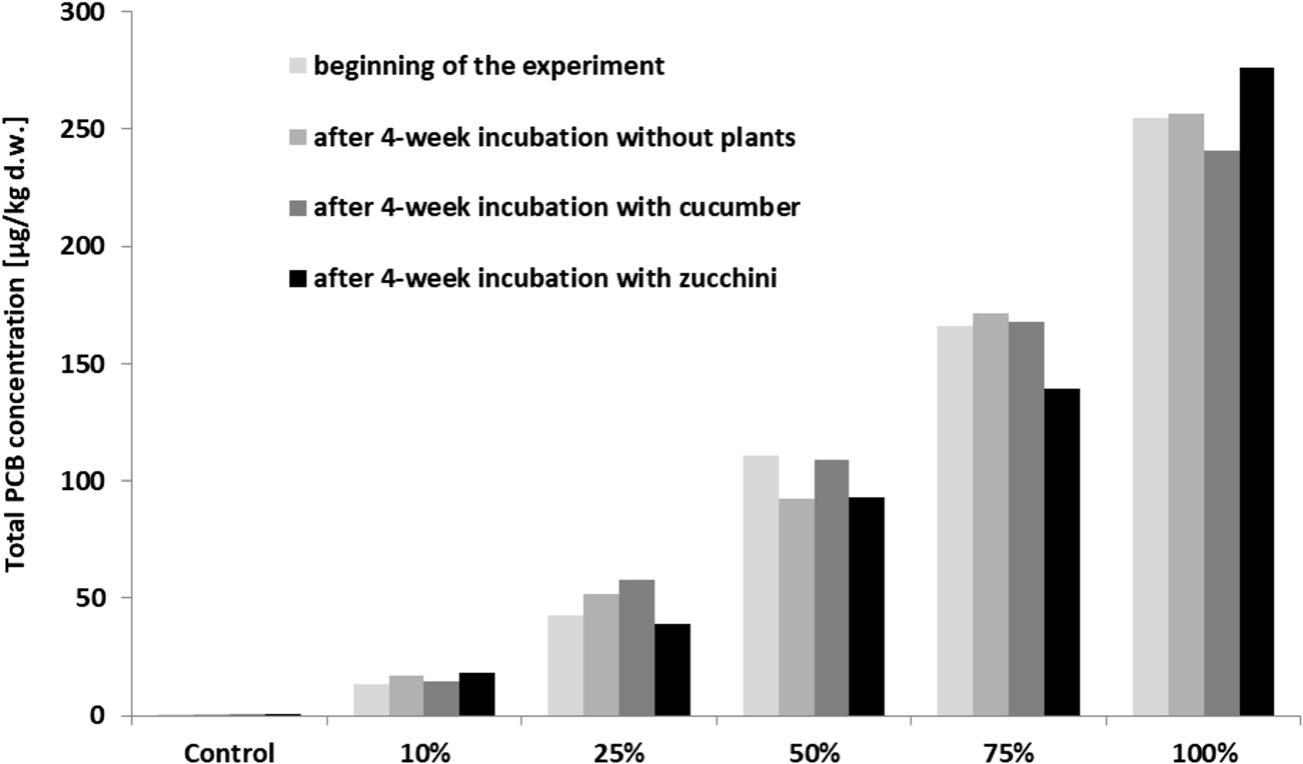
Concentrations of PCBs in soil amended with different proportions of Hudson River sediment at the beginning of the experiment and after 4-week incubation: without plants, with cucumber, or with zucchini

In our case, amendment of vegetable potting soil with Hudson River sediments led to a gradual increase in PCB content of the admixture/substratum: 0.410 μg/kg in control soil, 13.7 μg/kg when 10% of sediment was amended, 42.6 μg/kg for 25% sediment, 111 μg/kg for 50% sediment, 166 μg/kg for 75% sediment, and 255 μg/kg for 100% of sediment admixture (Fig. 1). Two-way analysis of variance showed that the content of PCBs in the growth medium was significant and did not depend on the variant of the experiment (unplanted and planted variant); however, our findings confirm that doses of bottom sediment have a strong effect (Table 2).

**Table 2:** Two-way analysis of variance of the effects of (A) sediment dose (0, 10%, 25%, 50%, 75%, 100%) and (B) experimental variant (i.e., unplanted or planted variant) on the total PCB content of the growth medium

Effect	MS	*F*	*p*
Dose of the bottom sediment (A)	**38,396.9**	**291.179**	**0.000000**
Variant of the experiment (B)	18.8	0.143	0.712354
A × B	44.7	0.339	0.879811
Error	131.9		

Over the 4-week cultivation period, zucchini planted samples demonstrated the most pronounced, however statistically unconfirmed, decrease in soil PCB content. In this case, an 8% decline in total PCB content was observed in the treatment with 25% sediment admixture; in contrast, a 16% decrease was observed for the treatments amended with 50% and 75% sediment (Fig. 1). For cucumber, amendments of 10%, 25%, and 75% sediment demonstrated a slight increase in total PCB content, whereas 50% and 100% sediment amendments showed a 2% and 6% decline in total PCB content, however again statistically not confirmed (Fig. 1).

The concentrations of the main PCB homologues are presented in Table 3, and the corresponding reductions in relation to the initial values are given in Table 4. Among the PCB homologues, the highest concentrations were noted for the lower chlorinated di-, tri-, and tetra-CB congeners, which amounted to 82–85% of the total PCB content. Such high predominance may indicate that light Aroclors (1242 and 1248) may be the main PCB source in the Hudson River sediments (Cho et al. 2001; Rodenburg and Ralston 2017). Four-week incubation led to changes in the concentrations of select PCB-homologue groups. The greatest reduction in concentrations was observed for zucchini (30 samples with a reduction ranging from 1% to 88%), followed by cucumber (24 samples with a reduction ranging from 2% to 88%) and the no-plant variant (23 samples with a reduction of 1% to 88%) (Table 4). It is important to underline that the highest reductions of 88% were calculated based on LOQ value of 0.05 μg/kg and were observed only in the case of control samples. Among other variants, zucchini planted samples showed the highest reductions being as high as 83%, followed by cucumber variants—with the highest observed reduction of 58%, and unplanted samples—with the reductions up to 30%. What is more, the highest reductions were noted for higher chlorinated PCB-homologue groups: hepta- and octa-PCBs (Table 4).

**Table 3:** Concentrations of PCB families in soil amended with different proportions of Hudson River sediment at the beginning of the experiment and after 4-week incubation: without plants, with cucumber, or with zucchini

PCB-congener families	Di-CBs	Tri-CBs	Tetra-CBs	Penta-CB	Hexa-Cbs	Hepta-CBs	Octa-CBs	Nona-CBs	Deca-CB	Total
Variants	Beginning of the experiment									
Control	0.41	< LOQ	< LOQ	< LOQ	< LOQ	< LOQ	< LOQ	< LOQ	< LOQ	**0.410**
10%	2.60	< LOQ	6.81	2.59	1.20	0.270	0.260	< LOQ	< LOQ	**13.7**
25%	5.89	13.6	15.41	4.49	2.09	0.680	0.290	0.070	0.060	**42.6**
50%	17.3	30.0	44.75	10.8	5.35	1.56	0.660	0.250	0.090	**111**
75%	25.0	50.6	64.27	15.4	6.85	2.55	0.750	0.490	0.170	**166**
100%	43.5	87.2	80.6	26.8	10.6	3.86	1.45	0.490	0.210	**255**
After 4-week incubation without plants										
Control	< LOQ	< LOQ	< LOQ	< LOQ	0.690	< LOQ	0.110	< LOQ	< LOQ	**0.790**
10%	2.27	4.06	6.56	2.42	1.26	0.190	0.210	< LOQ	< LOQ	**17.0**
25%	7.10	17.0	18.4	5.36	2.17	0.780	0.590	< LOQ	< LOQ	**51.4**
50%	13.7	23.4	35.0	12.6	5.32	1.40	0.530	0.320	0.110	**92.4**
75%	27.6	55.2	60.4	16.8	7.34	2.74	0.830	0.450	0.150	**171**
100%	41.5	88.3	87.5	23.7	9.56	3.82	1.06	0.490	0.190	**256**
After 4-week incubation with cucumber										
Control	< LOQ	< LOQ	0.33	< LOQ	0.25	< LOQ	< LOQ	< LOQ	< LOQ	**0.570**
10%	2.18	3.88	5.58	1.92	0.960	0.300	0.110	< LOQ	< LOQ	**14.9**
25%	7.37	20.4	21.5	5.04	2.22	0.820	0.230	0.070	0.050	**57.8**
50%	16.5	35.1	38.8	10.3	4.65	1.80	0.580	0.670	0.230	**108.7**
75%	25.6	55.5	59.7	16.1	7.14	2.50	0.870	0.390	0.180	**168**
100%	41.1	79.7	83.0	22.2	9.19	3.57	1.00	0.460	0.180	**240**
After 4-week incubation with zucchini										
Control	< LOQ	< LOQ	0.17	< LOQ	0.24	< LOQ	< LOQ	< LOQ	< LOQ	**0.410**
10%	2.62	5.34	6.94	1.87	1.33	0.250	< LOQ	0.080	< LOQ	**18.4**
25%	5.81	13.4	13.5	3.67	1.84	0.680	< LOQ	0.130	< LOQ	**39.0**
50%	15.2	31.7	30.7	8.73	3.74	1.81	0.380	0.360	0.100	**92.8**
75%	22.5	45.3	47.4	14.1	6.42	1.94	1.23	0.300	0.140	**139**
100%	47.2	86.1	102.2	25.6	9.30	4.45	0.540	0.460	0.190	**276**

**Table 4:** Reductions (%) in the concentrations of PCB families after 4-week incubation (without plants, with cucumber, or with zucchini) in comparison to values determined at the beginning of experiment

PCB-congener families	Di-CBs	Tri-CBs	Tetra-CBs	Penta-CB	Hexa-Cbs	Hepta-CBs	Octa-CBs	Nona-CBs	Deca-CB	Total
After 4-week incubation without plants										
Control	88*	0*	0*	0*	− 1280*	0*	− 120*	0*	0*	− 93
10%	13	− 8020*	4	7	− 5	30	19	0*	0*	− 24
25%	− 21	− 25	− 20	− 19	− 4	− 15	− 103	29*	17*	− 21
50%	21	22	22	− 17	1	10	20	− 28	− 22	17
75%	− 10	− 9	6	− 9	− 7	− 7	− 11	8	12	− 3
100%	4	− 1	− 9	12	10	1	27	0	10	− 1
After 4-week incubation with cucumber										
Control	88*	0*	− 560*	0*	− 400*	0*	0*	0*	0*	− 39
10%	16	− 7660*	18	26	20	− 11	58	0*	0*	− 9
25%	− 25	− 51	− 40	− 12	− 6	− 21	21	0	17	− 36
50%	4	− 17	13	4	13	− 15	12	− 168	− 156	2
75%	− 2	− 10	7	− 4	− 4	2	− 16	20	− 6	− 1
100%	5	9	− 3	17	14	8	31	6	14	6
After 4-week incubation with zucchini										
Control	88*	0*	− 240*	0*	− 380*	0*	0*	0*	0*	0
10%	− 1	− 10,580	− 2	28	− 11	7	81*	− 60*	0*	− 34
25%	1	2	12	18	12	0	83*	− 86	17*	8
50%	12	− 6	31	19	30	− 16	42	− 44	− 11	16
75%	10	10	26	8	6	24	− 64	39	18	16
100%	− 9	1	− 27	5	13	− 15	63	6	10	− 8

*Calculated based on LOQ value of 0.05 μg/kg; negative values indicate increased concentration in relation to initial values at the beginning of experiment; positive values (grey) indicate reduction in relation to initial values at the beginning of experiment

Plants have been shown to remove POPs from soils (Zhao et al. 2006; Susarla et al. 2002; Macek et al. 2000). However, our results indicate that only in the case of zucchini slight reduction in total PCB content in soil–sediment medium was observed. Urbaniak et al. (2016) reported that cultivation of zucchini (*Cucurbita pepo* cv. Atena Polka) in soil amended with sewage sludge reduced the concentrations of dioxins by 37% and decreased toxic equivalents (TEQ) by 68%. Similarly, Wyrwicka et al. (2014) reported that cucumber reduced PCB content in sludge and sediment amended soil by 38.6% and 27.4%, respectively, after 5 weeks of cultivation. It is important to note that phytoextraction capacities against organic compounds can vary between species and cultivars. Urbaniak et al. (2017a) found that zucchini was more effective in dioxin removal (37% reduction) than cucumber (24% reduction). Inui et al. (2008) found that among three tested *C. pepo* cultivars, ‘Black Beauty’ and ‘Gold Rush’ demonstrated 180-fold greater accumulation of organic compounds than ‘Patty Green.’ Moreover, plants, particularly cucurbits, play an important role in PCB degradation due to a symbiotic relationship with plant growth-promoting bacteria. This symbiosis is related to a range of plant exudates released into the soil–root zone. Plant exudates not only support co-metabolic degradation processes by stimulating the proliferation and activity of PCB-degrading bacteria but also act as biostimulants that increase cell mass in the rhizosphere. Terzaghi et al. (2019), for example, demonstrated the positive role of long-term rhizostimulation in driving the modifications among soil microbiota, leading to its higher enzymatic activity and thus removal capabilities against PCBs. The authors showed that pumpkin (*Cucurbita pepo* ssp. *pepo*) is effective in reducing the concentration of PCBs; however, the most efficient removal is when it is co-cultivated with tall fescue and *Rhizobium* spp. and mycorrhizal fungi.

The plant exudates can also contain bio-surfactant molecules that play a crucial role in improving the bioavailability and degradation of PCBs (Vergani et al. 2017). The lack or low removal of PCBs from sediments may be related to several factors including poor bioavailability of PCBs to plants. Moreover, the bacterial metabolism of PCB congeners may lead to the production of chlorobenzoates, dihydrodiols, dihydroxy-biphenyls, and other dead-end metabolites that exhibit toxic properties to microorganisms, affecting cell viability and thus inhibiting the entire PCB degradation process (Dai et al. 2002; Passatore et al. 2014). Consequently, the recalcitrance of PCBs to the applied remediation techniques and the low efficiency of the degradation process may be explained by the toxicity of certain metabolites generated during the PCB-transformation processes (Passatore et al. 2014).

It needs to be underlined that contaminated environmental matrices such as Hudson River sediments are mixtures not only of different congeners of PCBs, which differ with regard to their degradation pathways and intermediate degradation products, but they also include a range of other organic compounds (dioxins, polycyclic aromatic hydrocarbons) that may influence remediation efficiency. Consequently, the ongoing transformation processes may elicit several inhibition effects that in turn affect the efficiency of the remediation process (Dai et al. 2002; Passatore et al. 2014).

The relatively short incubation period used in the study may have an effect on our results. Tu et al. (2017) demonstrated that longer incubation times were associated with greater removal of PCB-77, with 62%, 73%, and 91% removal observed after 45, 75, and 105 days of incubation. This increasing removal rate was also found to be associated with growing number of PCB-degrading bacterial genes, at 187%, 279%, and 483% of the initial soil after 45, 75, and 105 days of incubation, respectively. Liang et al. (2014) report 40% and 29.5% removal of the total PCB content in switchgrass planted and unplanted soil after 24 weeks (168 days). Kurzawova et al. (2012) demonstrated 40% and 25% PCB removal after 3-month incubation (90 days) with nightshade and tobacco, respectively. These findings suggest that both experiment duration and choice of suitable plant cultivars play key roles in the efficient removal of PCBs. With this in mind, extension of the incubation time could accelerate PCB degradation and enhance remediation efficiency.

### The effect of sediment and cucurbit growth on phytotoxicity

One of the fast ecotoxicological tests available for assessing overall toxicity of a solid matrix (soil/sediment) is the Phytotoxkit bioassay. Phytotoxicity is defined as the presence of detrimental effects on various physiological processes of plants, such as seed germination and plant root elongation, caused by specific substances and general physico-chemical conditions present in the test matrix.

In this study, phytotoxicity of soil samples amended with the sediment collected from Hudson River was assessed using *L. sativum* as the test species. This plant species was selected based on our earlier studies (Urbaniak et al. 2016, 2017a, b), as well as those performed worldwide (Oleszczuk and Hollert 2011; Oleszczuk et al. 2012; Ramirez et al. 2008a, b), that demonstrate it greatest sensitivity and suitability for assessing phytotoxicity of soil and sediments.

The soil–sediment mixtures were not found to have any toxic properties (inhibition of root length lower than 20%) at the beginning of the experiment, nor after growth of cucumber and zucchini and natural attenuation (no plants). At the beginning of the experiment, all soil–sediment mixtures were found to stimulate *L. sativum* root growth, ranging from − 6% to − 38%. Nevertheless, this stimulation decreased over the 4-week incubation period. The greatest inhibition was observed with sediment mixtures that did not have plants grown in them (inhibition between 6% and 10%) and for zucchini planted samples (from 2% to 19%) (Table 5). Soil–sediment mixtures grown with cucumber demonstrated high stimulation of *L. sativum* root growth, being − 27%, − 28%, − 20%, and − 11% for 0%, 10%, 25%, and 100% of sediment admixture (Table 5). An admixture of 50% and 75% sediment led to slight inhibition of *L. sativum* root length, the degree of reduction being 11% and 1%, respectively; similarly, minor inhibition was noted for the no-plant variant (inhibition of 6% and 10%). Nevertheless, as none of the inhibition values obtained for cucumber variants exceeded the 20% threshold, the soil–sediment mixtures grown with this plant species cannot be regarded as toxic (Table 5). Soil–sediment admixtures taken from both plant species displayed significant differences in phytotoxicity between the beginning and the end of the 4-week cultivation period. Treatments with cucumber presented statistically significant differences in phytotoxicity from those planted with zucchini (Table 5).

**Table 5:** Phytotoxicity of samples measured using *L. sativum* as a test plant; calculated in reference to OECD soil

Sediment dose	Beginning of the experiment	After 4-week cultivation period		
		No plant	Cucumber	Zucchini
Control	− 31^a^	− 6^a^	− 27^a, c, d^	14^b, c,d^
10%	− 18^a^	8^b^	− 28^a, c, d^	7^b, c, d^
25%	− 6^a^	8^b^	− 20^a, c, d^	− 2^a, c, d^
50%	− 38^a^	6^b^	11^b, c, d^	2^b, c, d^
75%	− 31^a^	10^b^	1^b, c, d^	10^b, c, d^
100%	− 20^a^	− 22^a^	− 11^a, c, d^	19^b, c, d^

No asterisk—statistically non-significant results

^a^Negative values indicate stimulation of *L. sativum* root growth in comparison to uncontaminated reference soil (OECD soil derived with the Phytotoxkit test)

^b^Positive values indicate inhibition of the *L. sativum* root growth in comparison to uncontaminated reference soil (OECD soil derived with the Phytotoxkit test)

^c^Statistically significant in comparison to the beginning of the experiment

^d^Statistically significant in comparison to no plant variant after 4-week incubation

The stimulation of *L. sativum* root growth observed in sediment-amended soil could possibly be due to the composition of the amendment and good water retention capacity of soil–sediment treatments. The Hudson River sediment was rich in P and Fe (Table 1). These properties alleviate the toxic effects of PCBs and other pollutants, which manifest themselves in increased *L. sativum* root growth. The decrease in the stimulation observed in unplanted soil can be related to leaching of macronutrients from soil–sediment mixture during the incubation period: the pots were systematically watered to keep the experimental conditions same with planted treatments during the entire incubation time. Only 100% sediment maintained the same level of stimulatory effect; however, this medium was characterized by a dense sediment structure, preventing the leaching of macroelements. Moreover, the presence of high TOC in the soil may influence PCB behavior in the prepared soil–sediment mixtures; according to Terzaghi et al. (2018a), organic carbon, especially in a dissolved form, not only increased PCB bulk water concentration acting as a “spoon feeder” for bacteria but also enhanced PCB degradation through mediating the infiltration. Consequently, different proportions of soil, rich in TOC, and sediments, characterized by 22-fold lower TOC content, could have an impact on the soil parameters such as PCB content and phytotoxicity.

Different patterns were observed for treatments with plants, with significantly higher stimulation observed for cucumber than zucchini (Table 5). Such variation can be related to species-specific properties, as well as differences in the activity of soil–sediment microbiota under the influence of host cucurbits as plant nutrient preference influences the activity of rhizosphere microbiome. Cai et al. (2017) found that cultivation with cucumber resulted in a different soil microbiota profile than tomato cultivation. Poli et al. (2016) also presented the role of plant genotype on rhizosphere microbiota. Consequently, species-specific nutrient preferences and species-dependent root exudates may influence the overall phytotoxicity of samples through their influence on soil properties and soil–sediment microbial activity.

### The effect of sediment application on cucurbit growth and biomass

Although several studies concerning the environmental impact of PCBs as well as PCB-contaminated sediments, including Hudson River sediments, exists in the literature, these studies mainly address contamination levels in situ and the effects of PCBs on animals such as fish (Field et al. 2016; Pinkney et al. 2017; Maceina and Sammons 2015), birds (Madden and Skinner 2016; Baker et al. 1976; Foley 1992; Kim et al. 1984, 1985; O’Keefe et al. 2006), or invertebrates (Cho et al. 2004). In contrast, our study examines the effect of sediments on plants. Whereas the abundance of organic matter and nutrients, especially P, makes sediments a good source of nutrients, the presence of organic pollutants may place stress on plants. Our findings demonstrate the varying responses of cucurbits to the application of Hudson River sediments.

**Fig. 2:**
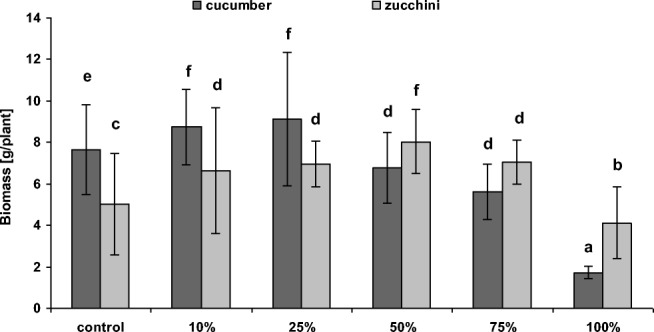
Mean (± SD) fresh aboveground biomass of cucumber and zucchini (after 4 weeks) cultivated in soil amended with different proportions of Hudson River sediments. Error bars indicate standard deviation (*n* = 9). Letters denote a significant difference with *p* > 0.05 (the Tukey post hoc test)

**Fig. 3:**
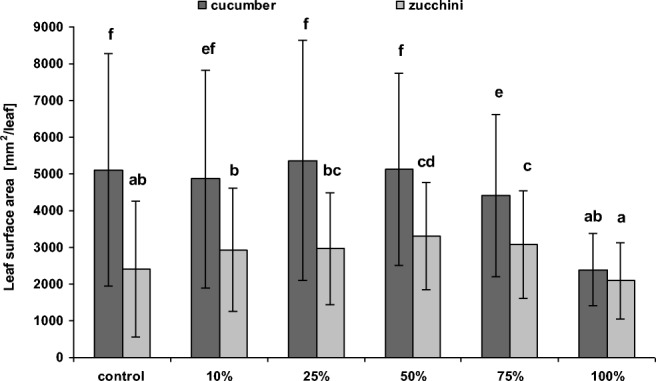
Mean (± SD) leaf surface area of cucumber of zucchini cultivated in soil (after 4 weeks) amended with different proportions of Hudson River sediments. Error bars indicate standard deviation (*n* values for cucumber—control = 32; 10% = 42; 25% = 41; 50% = 38; 75% = 32; 100% = 16; *n* values for zucchini—control = 31; 10% = 32; 25% = 41; 50% = 44; 75% = 41; 100% = 34). Letters denote a significant difference with *p* > 0.05 (the Tukey post hoc test)

**Fig. 4:**
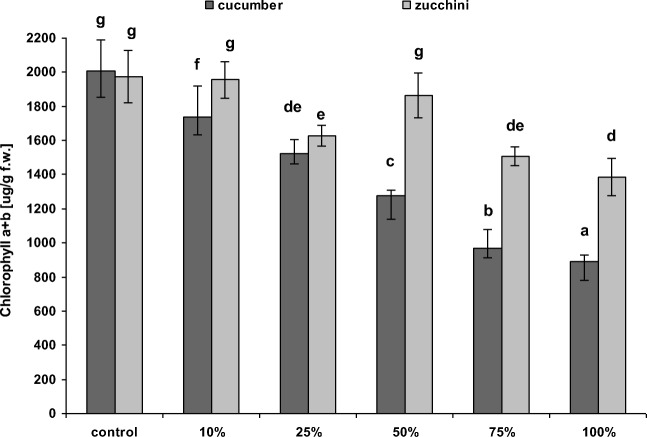
Mean (± SD) chlorophyll *a* + *b* content in the leaves of cucumber and zucchini (after 4 weeks) cultivated in soil amended with different proportions of Hudson River sediments. Error bars indicate standard deviation (*n* = 9). Letters denote a significant difference with *p* > 0.05 (the Tukey post hoc test)

Our results indicate that cultivation of cucurbits on soil amended with sediments from the Hudson River near Waterford, especially at lower doses, significantly increased fresh biomass compared to controls (i.e., 0% sediment). However, the response of plants to PCB-contaminated sediments was significantly dependent on both species and dose. In the case of cucumber, only the 10% and 25% sediment admixtures led to significant higher aboveground biomass production (114% and 126% of the control, respectively). Higher doses of 50%, 75%, and 100% sediment admixtures significantly reduced aboveground biomass production in comparison to control (89%, 73%, and 23%, of the control, respectively) (Fig. 2). In the case of zucchini, the sediments appeared to have a positive influence on aboveground plant biomass, which was found to significantly increase with the proportion of sediment: this increase was found to be 132%, 138%, 160%, and 140% of control values for 10%, 25%, 50%, and 75% sediment admixtures, respectively; only 100% sediment admixture caused significant lower zucchini aboveground biomass (82% of the control) (Fig. 2). The results also demonstrated a significant increase in the zucchini leaf surface area in variants amended with 50% and 75% sediment admixtures (Fig. 3). For cucumber, in turn, 10%, 25%, and 50% sediment admixtures did not reveal significant changes in the leaf surface area, while application of higher doses led to significant decrease in the leaf surface area in comparison to control plants (Fig. 3). However, correlation analyses confirmed a significant positive correlation between leaf surface area and biomass of both plants. An increase in plant biomass following sediment amendment was also reported by Karak et al. (2013) for mustard. However, no correlation was found between an increased biomass and greater leaf surface area with the production of photosynthetic pigments by zucchini; this stands in contrast with cucumber, where such correlations have been recorded (Fig. 4, Table 6).

**Table 6:** Relationships between content of PCB in the soil–sediment mixtures and the biometric and physiological parameters of the cucumber and zucchini grown therein

Parameter	Cucumber			Zucchini		
	PCB	Biomass	Leaf surface	PCB	Biomass	Leaf surface
Biomass	− 0.905*			− 0.414		
Leaf surface	− 0.826*	0.940*		− 0.432	0.997*	
Chlorophyll	− 0.964*	0.768*	0.677*	− 0.845*	0.339	0.323

*Significant at *p* ≤ 0.05

The application of sediments led to significantly lower production of chlorophyll *a* + *b* by both plants. However, cucumber was more sensitive to the sediment than zucchini and demonstrated lower production of photosynthetic pigment with regard to 10%, 25%, 50%, 75%, and 100% sediment amendment: 87%, 76%, 63%, 48%, and 44% of control values for cucumber, and 99%, 82%, 94%, 76%, and 70% for zucchini (Fig. 4).

Correlation analyses also found cucumber to display greater sensitivity, wherein a higher number of statistically significant dependences between PCB content and biometric and physiological parameters were obtained for cucumber than zucchini (Table 6). While the correlation between total PCB concentration and plant parameters was generally negative for both tested species, for zucchini, only chlorophyll content was significantly negatively correlated with total PCB concentration in the growing medium. Regardless of the species, the highest values of the correlation coefficient were observed between chlorophyll *a* + *b* and PCB content (Table 6). This suggests that chlorophyll *a* + *b* measurement is a suitable indicator of plant stress on PCB concentration in the growing medium. Wyrwicka et al. (2019) report that total chlorophyll content, together with chlorophyll *a*/*b* ratio, were good indicators of the initiation of plant senescence and changes in the biochemical processes in cucumber plants following the application of PCB-contaminated sewage sludge and urban sediment. Reduced chlorophyll content is one of the first symptoms of plant senescence, which is connected with the breakdown of thylakoid membranes and the degradation of thylakoid-bound proteins; hence, chlorophyll content have previously been used as indicators of plant aging (Prochazkova et al. 2001; Nath et al. 2013; Gomes et al. 2016).

## Conclusions

Our findings revealed a complex nature of Hudson River sediment for its potential use as a growth medium after amendments with soil. Although sediment represents a valuable source of P, Fe, and Ca, it is contaminated with PCBs that exceed TEC. Thus, soil-sediment mixtures can result in diverse responses with regard to phytotoxicity and the health of plants grown therein. PCB content of soil increased following amendment with sediment. However, sediment admixture stimulated *L. sativum* root growth at the beginning of the experiment. After 4-week cultivation period, zucchini grown soil–sediment substratum demonstrated slight inhibition of *L. sativum* growth, whereas cucumber grown substratum stimulated root growth of *L. sativum* compared to controls. The biomass of cucumber was lower than that of control at almost all applied doses of sediment. Zucchini showed increased biomass in all soil–sediment mixtures except for 100% sediment. Dose- and species-dependent responses were observed for plant leaf surface area being significantly higher for zucchini amended with 50% and 75% of sediment. Cucumber, in turn, showed a reverse trend with significantly lower leaf surface area for 50% and 75% sediment admixture. Chlorophyll content of cucumber and zucchini decreased with increasing sediment doses. Zucchini was found to be more resistant than cucumber to sediment amendment of soil. Moreover, zucchini planted samples showed higher, however statistically unconfirmed, reduction of the total PCBs than cucumber and unplanted samples following the 4-week incubation period. The degree of remediation could well be affected by extending the duration of the experiment, as well as application of other cucurbit cultivars. Further studies are intended to evaluate the accumulation of PCBs in tissues of cultivated plants and to assess of the fate of the pollutants under environmental conditions instead of controlled laboratory conditions.
